# PrEditR: A protein-centric platform for CRISPR-mediated base editor sgRNA design

**DOI:** 10.64898/2026.05.15.725600

**Published:** 2026-05-16

**Authors:** Felipe Vasquez-Castro, Leonardo D. Sanchez Solis, Samuel A. Myers

**Affiliations:** 1 Laboratory for Immunochemical Circuits, La Jolla Institute for Immunology, La Jolla, CA 92037, United States of America; 2 Bioinformatics and Systems Biology Ph.D. Program, University of California, San Diego, La Jolla, CA, 92093, United States of America; 3 Center of Autoimmunity and Inflammation, La Jolla Institute for Immunology, La Jolla, CA 92037, United States of America; 4 Department of Pharmacology, University of California, San Diego, La Jolla, CA 92037, United States of America; 5 Moores Cancer Center, UCSD Health, La Jolla, CA 92037, United States of America

**Keywords:** Proteomics, Functional genomics, Signal transduction, Post-translational modifications

## Abstract

**Motivation:**

Post-translational modifications (PTMs) are critical to protein function, yet the function of most known modification sites remains uncharacterized. CRISPR-mediated phenotypic screens using base editors offer a powerful approach to dissecting PTM function at scale. However, existing sgRNA design tools for base editing applications are DNA-centric and lack the throughput required to integrate seamlessly with mass-spectrometry-based proteomics experimental outputs.

**Results:**

We introduce protein editing in R, PrEditR, an open-source, protein-centric tool for high-throughput sgRNA design for custom base editor screens. PrEditR enables users to designate specific amino acid residues in proteins and design protospacer sequences to target the endogenous gene to install missense mutations via base editors.

**Availability and Implementation:**

PrEditR is available on GitHub and Docker Hub.

## Introduction

Post-translational modifications (PTMs) control nearly every aspect of cellular and organismal biology. Although mass spectrometry-based methods have mapped tens of thousands of chemically modified amino acid side chains across the proteome, our understanding of their roles and functions remain undetermined for more than 98% of the more than a quarter million reported.^1^

CRISPR-mediated base editors offer a promising genetic engineering tool to address this gap. These tools expand the scope of genome editing by fusing a nickase Cas9 protein with specific nucleotide deaminase enzymes. Fusing Cas9 with a cytosine deaminase (e.g., rAPOBEC1) creates a Cytosine Base Editor (CBE), which drives cytosine-to-thymine (C-to-T) transitions. Conversely, fusion with an adenine deaminase (e.g., TadA) creates an Adenine Base Editor (ABE), resulting in adenine-to-guanine (A-to-G) transitions.^2,3^ Because base editors are built from Cas9 proteins, both CBEs and ABEs are programmable through the protospacer sequence in the single guide RNAs (sgRNAs) and are highly suitable for large-scale phenotypic screens. By designing a screen that specifically targets nucleotides encoding post-translationally modified amino acids, i.e. “knocking out” the PTM site without affecting transcriptional regulatory elements, base editors can be used to test the functional relevance of PTM sites in a high-throughput manner.^4,5^

Currently available tools to design sgRNAs for base editing are mostly DNA-centric, requiring users to provide a DNA sequence to edit instead of indicating a specific amino acid residue position in a given protein. Some are exclusively web-based, which limits their integration into automated pipelines.^6,7^ On the other hand, BEscreen^8^ allows the targeting of specific residues. However, it requires users to specify the resulting mutation a priori (e.g., GAPDH-L270F); if the requested mutation is not feasible, the tool fails to generate a sgRNA of interest. Many proteins show a high degree of tolerance to random mutations, and in PTM functional screens, the identity of the substituted amino acid is often less important than the loss of the modifiable residue itself.^4,9^ To address these limitations, we developed PrEditR (pronounced “preditor”): an R-based tool for protein-centric sgRNA design for custom, reproducible base editing applications. Unlike BEscreen, PrEditR only requires users to input the target residue and not the resulting mutation, and it will produce all sgRNAs that target such residue as well as the resulting mutations for each of them. To meet the growing volume of data generated in proteomics experiments, the tool supports parallel execution and is scalable across computing environments, from personal laptops to high-performance clusters (HPCs).

## 2 Implementation

### 2.1 Framework

PrEditR is written in R and containerized using Docker to ensure seamless cross-platform deployment. The Docker image is available for amd64 and arm64 architectures and offers both a command-line interface (CLI) and a Shiny-based graphical user interface (GUI) in the same image. To mitigate performance bottlenecks associated with server-based requests, PrEditR comes with the human (hg38) and mouse (mm10) genome assemblies and genomic features annotations built-in. Optionally, users can provide a Bowtie^10^-indexed genome (in .ebwt format) to perform protospacer off-target searches. Parallel processing is supported via the future R framework^11^, allowing for computational scalability.

### 2.2 Core Logic

PrEditR leverages the crisprVerse^12^ ecosystem but extends it by accurately converting relative absolute genomic positions to protein-based coordinates (e.g., chr10:80786010–8078601 to valine 218 in transcript ENST00000264657). Considering that base editors typically have narrow optimal editing windows of around 4–10 bp,13 this mapping must be precise and account for transcript structure, splicing, and gene strand orientation as base editing can occur directly on the coding strand or indirectly via the opposite strand. Equally important, the resulting edited genomic sequence must be mapped back, in-frame, to the protein sequence to evaluate potential bystander edits as the editing window can span codons from adjacent amino acids.

### 2.3 Configuration

**Figure 1.**
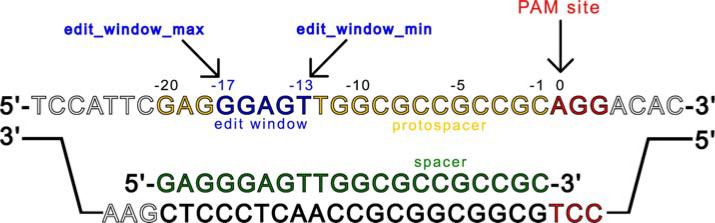
Diagram of the convention used in PrEditR. The PAM (protospacer adjacent motif) site is assigned position 0. The editing window is determined by the parameters edit_window_min and edit_window_max values, which represent the closest and furthest positions upstream (5’) of the PAM in nucleotides. As denoted in Section 6 (Limitations and Troubleshooting), PrEditR assumes that the PAM sequence lies immediately downstream (3’) of the protospacer.

The PrEditR workflow is driven by two mandatory CSV configuration files that define the target amino acids and the specific parameters of the base editors used. The first of these, the Base Editor Configuration (editors.csv), defines the properties of the base editors utilized for guide design and allows for multiple editors to be used in a single run. Each entry in this file requires a unique name as an identifier, the PAM sequence recognized by the nuclease (e.g., NGG, NG, NGN, …), and the protospacer length of the gRNA, which is typically 20 nucleotides. Additionally, this file must specify the specific nucleotide conversion (denoted as edit_type; e.g., “a2g” for an ABE) and the editing window through the edit_window_min and edit_window_max values, which represent the closest and furthest positions upstream of the PAM in nucleotides, respectively (Fig. 1)

The second required file, the Targeting Specifications (targets.csv), details the amino acid targets and the specific base editors intended to modify them. Because the system is isoform-aware, target proteins must be identified using their corresponding Ensembl transcript or UniProt IDs. While the official gene symbol can be included, it is optional and will be automatically populated by PrEditR if left blank. For each target, the file must specify the single-letter amino acid of the target residue and its numerical position within the protein sequence. Finally, the editor column must contain a name that exactly matches one of the unique identifiers defined in the editors.csv file to ensure the correct parameters are applied to the target site. Templates for these files are provided in Supplementary Tables 1 (targets.csv) and 2 (editors.csv), as well as the corresponding output example in Supplementary Table 3.

When the most suitable base editor for a specific target or screen is unknown, users are encouraged to leverage PrEditR’s ability to accommodate unlimited different editors in a single run. For N different editors, each target must be defined N times in targets.csv, one for each different editor.

### 2.4 Performance

To ensure PrEditR remains accessible to users without HPC resources, we optimized the platform to balance memory consumption and runtime across both small- and large-scale experiments. Parallel processing is driven by the future framework’s multicore strategy, which leverages process forking to efficiently share unmodified objects across workers. Because R’s native garbage collection inadvertently disrupted this shared-memory model and inflated the memory footprint, we mitigate this overhead by aggressively minimizing the parent environment prior to parallel execution.

**Figure 2.**
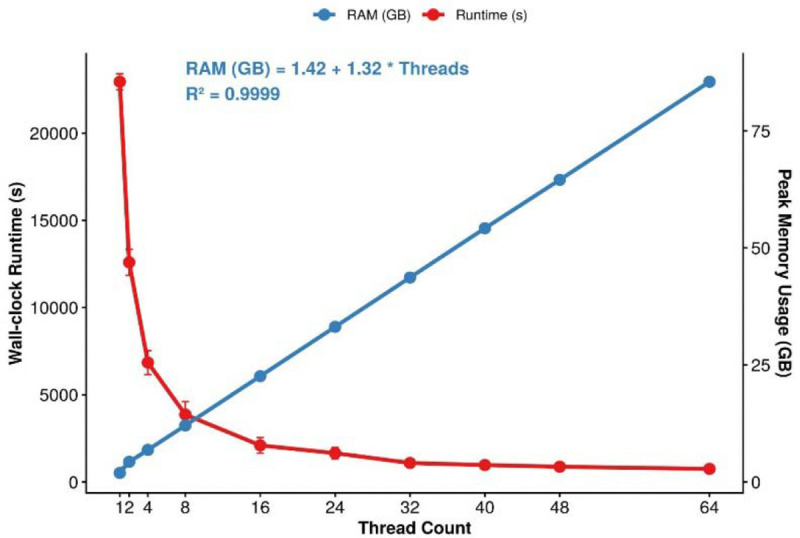
Peak memory utilization in gigabytes and wall-clock runtime in seconds by PrEditR in command-line mode against 9200 phosphorylation sites for different numbers of threads. Maximum memory utilization follows a linear trend with respect to thread counts. Wall-clock runtime is constrained by the number of physical cores in the hardware. Each point represents the average of 20 runs. The data used in this plot is in Supplementary Table 4.

To evaluate performance, PrEditR was run against the 9,200 unique phosphorylation site targets reported in Supplementary Table 2A of Kennedy et al.,4 using 1, 2, 4, 8, 16, 24, 32, 40, 48, and 64 threads, with 20 runs per number of threads. The analysis was conducted within a Singularity/Apptainer container on a system equipped with an AMD EPYC 7543P 32-core CPU (64 threads). As shown in Fig 2., peak RAM usage scales linearly with the number of threads while the wall-clock runtime does not benefit from additional threads beyond the number of physical cores, consistent with a CPU-bound workload reaching hardware parallelism limits. We hope this information will assist researchers in selecting an appropriate thread count to ensure efficient use of the computational resources available.

## 3 Use Cases

### 3.1 Functional Screening of Phosphorylation Sites

Kennedy et al.^4^ introduced a high-throughput approach to interrogate the functional relevance of phosphorylation sites and their control of NFAT transcriptional activity by integrating base editing with phenotypic screening. Their target selection was informed by upstream phosphoproteomic data derived from mass spectrometry, which provides a protein-centric readout, i.e., UniProt accession number and modified amino acid number for a specific isoform. However, their sgRNA design followed a DNA-centric workflow. Specifically, the authors employed a custom script to generate all possible sgRNAs for every gene represented in their dataset. Subsequent annotation of the resulting amino acid mutations allowed them to filter for sgRNAs targeting phosphorylation sites identified in their phosphoproteomic analyses. This workflow resulted in the unnecessary generation of numerous guides, as it designed guides for entire genes despite only requiring a small subset relevant to the phosphosites of interest. Moreover, their pipeline was dependent on real-time queries to the Ensembl server, which may present limitations in scalability, particularly in large-scale screens where multiple base editors must be evaluated to identify the most effective one.

To evaluate the performance and robustness of PrEditR, we benchmarked the tool against the 9,200 A-to-G unique phosphorylation sites from Kennedy et al.^4^ in Supplementary Table 2, which originally comprised 13,441 sgRNAs. Utilizing an AMD EPYC 7543P 32-core CPU with 24 threads, PrEditR successfully identified 11,451 sgRNAs (85.19%) by exact sequence identity (Supplementary Table 6). 1,978 sgRNAs (14.72%) were not found because the tool could not locate the target amino acid at the specified protein position. We cross-referenced the 1,978 missing sgRNAs with the original phosphoproteomics data from Supplementary Data 1 and confirmed that these discrepancies arose from mismappings between UniProt and Ensembl IDs. Specifically, for every failed guide represented in that subset, the Ensembl IDs reported in the sgRNA table did not correspond to the original UniProt IDs. For example, a site intended to mutate serine 716 on UniProt isoform Q9H8V3–1 (as dictated by the mass spectrometry data) was incorrectly mapped to Ensembl ENST00000232458 (isoform Q9H8V3–4). PrEditR correctly identified a threonine at that position on the mapped isoform and halted the design upon finding an inconsistency between the desired target amino acid and the actual amino acid for that specific isoform. This type of inconsistency is noted in the PrEditR output.

While the guides reported by the authors did target the desired genes, a fraction of them were incorrectly reported to different isoforms than the intended one. PrEditR is designed to be protein isoform-aware, incorporating multiple safeguards to ensure that individual mapping errors are handled safely and do not compromise large-scale runs. This use case highlights PrEditR’s utility as a critical bridge between mass spectrometry-derived proteomics and precise genomic engineering.

### 3.2 Functional Assessment of BRCA1 Variants

The base editor screen of BRCA1 variants by Kweon et al.^14^ serves as a good use case to demonstrate the significant workflow improvements offered by PrEditR when creating an amino acid-targeting mutational screen. The original method required manual retrieval of the large BRCA1 exon and intron-exon junction sequences. The 23 exons and their corresponding junctions with introns were fragmented into segments under 1000 bp for submission to Cas-Designer.^15^ PrEditR replaces this multi-step, laborious process with a single run to design guides targeting all amino acids in the protein.

We evaluated PrEditR against the 1,863 residues of BRCA1 (UniProt P38398–1; Ensembl ENST00000357654). Using 30 threads and a C-to-T editor, PrEditR identified 465 guides targeting 360 residues (Supplementary Table 5) in 225 seconds. While the original study did not provide the full output of their sgRNA search across the entire BRCA1 gene, comparison with their screened guides on Supplementary Table 1 (excluding sgRNAs on non-coding regions as PrEditR focuses exclusively on protein-coding regions) showed that PrEditR recapitulated all (100%) sgRNA sequences. Furthermore, PrEditR correctly reported the global translational effects of multiple base edits within single codons. For instance, in exon 23, where the original authors annotated individual G1803D (c.5408G>A) and G1803S (c.5407G>A) mutations, PrEditR accurately consolidated these adjacent edits into the resulting G1803N substitution.

The output from Cas-Designer lacked essential data for downstream analyses, such as specific genomic coordinates for edits and the resulting amino acid mutations, which had to be determined in additional, error-prone steps. PrEditR’s output automatically provides precise genomic coordinates, a summary of all amino acid mutations per guide (including bystander mutations), and the specific transcript targeted to remove ambiguity.

## 4 Validation

### 4.1 Stratified Validation Set Generation

To evaluate PrEditR’s accuracy and scalability, we designed ABE-NGG guides for 1,003 target protein-encoding genes using a base editing window of −13 to −17, resulting in an initial dataset of 974,003 target evaluations. This dataset encompassed both residues with successfully designed sgRNAs, and residues predicted to be untargetable. To construct a validation set that thoroughly tested PrEditR across diverse genomic contexts, we implemented a stratified sampling approach. We selected 72 unique target residues, taking one representative residue for every possible combination of four parameters: guide availability (0, 1, or multiple guides per residue), transcript region (first, second, or final third of the gene coding sequence), target strand orientation (coding or template), and exon boundary proximity (exon-internal, exon start edge, exon end edge, or split across an intron-exon junction). As such, we established a final validation set of 106 test cases, comprising 82 predicted sgRNAs and 24 instances where the tool identified a residue as untargetable, providing a comprehensive suite of edge cases to guide the refinement of the tool’s underlying algorithms.

### 4.2 Manual Benchmarking and Concordance

We performed manual sgRNA design for each target in the validation set using commonly utilized DNA-centric web tools. For each protein of interest, its relevant transcript was obtained from Ensembl and imported into Benchling. We utilized a 45-bp search window centered on the target residue to identify valid sgRNAs via the RGEN BE-Designer tool.^7^ For codons split across exons, we utilized dual search windows centered around each segment of the split codon to ensure the manual verification captured the full range of potential residue targeting. In addition, all editable bases within the base editing window were assumed to be edited.

This manual-guided debugging allowed us to resolve logic errors in complex genomic scenarios, ultimately achieving 100% concordance. PrEditR successfully identified all 82 valid sgRNAs, correctly predicted all 24 untargetable instances, and accurately annotated every resulting mutation across the entire validation set.

## 5 Discussion

Base editors have revolutionized the molecular biology toolbox by enabling targeted nucleotide conversions within a narrow window, allowing researchers to investigate PTM function at an unprecedented scale. However, a significant gap remains for reproducible, large-scale sgRNA design. PrEditR fulfills this need as a “one-stop” solution that streamlines sgRNA design while integrating critical experimental and analytical parameters often neglected by existing software.

Beyond simple design, the tool enhances experimental reliability by screening for downstream restriction enzyme sites and incorporating essential non-targeting and non-editing controls that are critical for establishing baselines for screens. To ensure results transparency, PrEditR provides exact genomic coordinates for every guide, which facilitates manual inspection and verification when necessary. Efficiency is further improved through an “append-to-input” output structure; by preserving and attaching results to the original input file, the tool allows researchers to include their own metadata that remains intact for downstream use.

To maximize accessibility, we developed PrEditR with both a command-line interface and an intuitive GUI. The software underwent extensive RAM optimization, democratizing access to researchers that lack high-performance workstations. Finally, by providing Docker images for both arm64 and amd64 architectures, we ensure that PrEditR is portable and accessible across the full spectrum of modern computing hardware.

## 6 Limitations and Troubleshooting

- PrEditR assumes that each base editor performs a single type of nucleotide mutation (e.g., A-to-G, C-to-T, C-to-G, …). For editors capable of multiple conversion types, a workaround is to define them as separate editor entries, one for each distinct conversion.
- Only DNA base editors are supported.
- PAM sequences are assumed to lie immediately downstream (i.e., 3’) of the protospacer (Fig 1.).
- All protospacers designed in a single run must be of the same length.
- PrEditR assumes uniform editing efficiency across all positions within the defined editing window; position-specific editing probabilities (i.e., weighted editing windows) are currently not supported.
- PrEditR comes with specific human (hg38) and mouse (mm10) genome versions built-in. To prevent server-querying-related bottlenecks, local UniProt to Ensembl IDs maps were constructed with the information on Ensembl.org as of November 25, 2025. These mappings might change over time, especially as unreviewed entries on UniProt are updated. Users can access the raw data and the scripts employed to construct the maps at /app/maps/<organism> in the Docker image. Researchers will be allowed to provide their own Ensembl-to-UniProt IDs maps to PrEditR in future versions.
- PrEditR queries local genetic databases using Ensembl IDs. If only a UniProt ID is provided, PrEditR will first map the UniProt ID to the corresponding Ensembl ID using the built-in database. For novel UniProt to Ensembl transcript ID associations (i.e., those identified after November 25, 2025), the local database might fail to find the corresponding Ensembl transcript ID provided only a UniProt ID. Providing the transcript Ensembl ID instead will circumvent this error.

## Supplementary Material

Supplement 1

## Data Availability

The source code for PrEditR is available on GitHub (github.com/fvasquezcastro/preditr) and distributed through Docker Hub (hub.docker.com/repository/docker/fvasquezcastro/preditr/general)
